# Association of Acetaminophen With Stevens-Johnson Syndrome and Toxic Epidermal Necrolysis: Pharmacologic Considerations and Treatment Options

**DOI:** 10.7759/cureus.41116

**Published:** 2023-06-28

**Authors:** Noah J Spillers, Patrick M Luther, Norris C Talbot, Gianni H Ly, Evan M Downs, Gabriel Lavespere, Denisa Pavlickova, Shahab Ahmadzadeh, Omar Viswanath, Giustino Varrassi, Sahar Shekoohi, Alan D Kaye

**Affiliations:** 1 Anesthesiology, Louisiana State University Health Sciences Center, Shreveport, USA; 2 Radiology, Louisiana State University Health Sciences Center, Shreveport, USA; 3 Pain Management, Valley Pain Consultants - Envision Physician Services, Phoenix, USA; 4 Pain Medicine, Paolo Procacci Foundation, Rome, ITA

**Keywords:** ten, antipyretic analgesics, adverse effects, stevens-johnson syndrome, acetaminophen

## Abstract

Acetaminophen is an extremely common drug with many implications for its analgesic and antipyretic properties. It has a unique mechanism of action and downstream effects that separate it categorically from non-steroidal anti-inflammatory drugs. These differences come with potential adverse effects that range from mild drug reactions to severe life-threatening emergencies. While acetaminophen's toxic liver effects are well known, a lesser-known adverse effect of this drug is its association with the development of Stevens-Johnson syndrome (SJS) and toxic epidermal necrolysis (TEN). These dermatological emergencies involve similar pathological processes, including apoptosis of the epidermis and sloughing of the dermis and mucosa from the underlying layers with a positive Nikolsky sign. Currently, SJS and TEN are considered immune-mediated type IV hypersensitivity reactions predominantly involving CD8+ T lymphocytes. Other immune mediators, including regulatory T cells, natural killer cells, interleukins, and drug metabolites are speculated to be involved, but their mechanisms have not been entirely determined. These conditions are differentially diagnosed by the percentage of body area affected with SJS and TENS, involving <10% and >30%, respectively. Genomic variations in human leukocyte antigens (HLA) genes have been implicated in the susceptibility and severity of acetaminophen-induced SJS/TENS, however, details of these interactions remain unclear. Acetaminophen’s widespread use and the morbidity of its associated skin pathologies SJS and TENS warrant an in-depth examination of the causative processes involved in their pathogenesis. It is critical that both physicians and patients be made aware that while acetaminophen is widely tolerated by most individuals, severe and potentially fatal interactions do occur, and further investigation is necessary to reduce these adverse effects.

## Introduction and background

The use of analgesics in daily life is commonplace within the United States. With as many as 52 million adults in the United States using acetaminophen-containing products per week, this medication has permeated over 600 therapeutics [1]. Acetaminophen, commonly referred to under the brand name Tylenol, has a global valuation of 9.8 billion USD as of 2022 [2]. Stabilized by a constant consumer base and high market valuation worldwide, acetaminophen is potentially one of the most influential over-the-counter (OTC) drugs of the current age. Although commonly referred to as non-steroidal anti-inflammatory drugs (NSAIDs), acetaminophen possesses little to no anti-inflammatory effects and is strictly effective as an antipyretic and analgesic. While acetaminophen is a high-profile OTC drug, the mechanism of action by which it operates is still under investigation.

Stevens-Johnson syndrome (SJS) and toxic epidermal necrolysis (TEN) are relatively rare diseases characterized by necrolysis and sloughing of the dermal layer. These diseases are pathophysiologically linked, and the diagnostic criteria for the two primarily differ in the percent body surface area affected. SJS affects <10% and TEN affects >30% of the body surface area and are considered overlapping conditions when 10-30% of the body surface area is implicated [3]. The most common precipitants of both conditions include anti-epileptics, NSAIDS, antibiotics, immune checkpoint inhibitors, and infection with mycoplasma pneumonia; however, in up to 30% of cases, no causative trigger can be identified. Around 9.2 million cases of SJS and 1.9 million cases of TEN are reported in the United States yearly, and both are considered dermatological emergencies with morbidity for SJS and TENS reported as 4.8-9% and 14.8-48%, respectively [3-5].

Acetaminophen-induced SJS and TEN cases have been reported via case reports in the past but make up a minority of known cases. Of these cases, all patients survived and responded to supportive intervention with long-term consequences reported in 13.9% of patients [6,7].

As the investigation continues, the association of acetaminophen usage with adverse effects such as SJS and TEN has become more widely recognized. When considering the widespread usage of acetaminophen, the implications of these effects become a more significant concern, denoting the need for further investigation to illuminate its contribution to the pathogenesis of these conditions.

Areas of uncertainty

As incidences of acetaminophen-induced SJS/TENS are relatively rare, the precise pathogenesis of the condition remains undetermined. Further analysis of the specific mechanism of action of acetaminophen and how it relates to the underlying immunological processes at play are required to garner a full understanding of the intermediaries involved. Furthermore, due to the widespread OTC use of acetaminophen and its use concomitantly with other pharmacologically active substances, previous cases of acetaminophen-induced SJS/TENS may have been misattributed to other causative factors or could be due to multifactorial pathogenesis in which both acetaminophen and other causative agents could simultaneously be implicated in the development of SJS/TENS. Additional meta-analyses and case reports are required to elucidate these intricacies and to shed light on the processes involved.

## Review

Data sources

This was a narrative review. The sources for this review are as follows: searching on PubMed, Google Scholar, Medline, and ScienceDirect; using keywords: Stevens-Johnson Syndrome, Acetaminophen, Toxic Epidermal Necrolysis, SJS, TEN. Additionally, other keywords included paracetamol, adverse effects, and paracetamol genomics. Sources were accessed between February 2023 and May 2023, and the literature search did not have any limitations for the time of publication.

Results

Clinical Implications of Acetaminophen Associated with SJS/TEN

SJS and TEN are characterized as drug-induced cutaneous eruptions and detachments that occur in the epidermis and mucosal membranes. SJS and TEN cases are rare, but they have a high mortality rate of 23% at six weeks [8]. In a retrospective study, the serious adverse events reported from acetaminophen in Europe were analyzed from 2007 to 2018. A total of 4,589 individuals were analyzed into different hypersensitivity reactions. Among the total individuals, 129 individuals were reported to have SJS, and 108 individuals with TEN [9]. Acetaminophen along with valdecoxib, lamotrigine, phenytoin, and furosemide have been shown to be associated with SJS and TEN as well. In 2013, the Food and Drug Administration (FDA) confirmed this association in rare instances [9]. Potential sequelae apart from the cutaneous and mucocutaneous lesions can include corneal scarring, irreversible respiratory damage, and other ocular surface reactions [8,10]. Here, we are interested in looking at the correlation between the ingestion of acetaminophen with the occurrence of SJS or TEN. Several studies show the linkage of acetaminophen intake with the development of SJS and other defects. Raising awareness of this connection is imperative within the medical community and can be pivotal in the prevention of drastic outcomes that could have been avoided during the prodromal phase of SJS/TEN, which is often mistaken for other febrile illnesses. In several case reports, there were many similarities to compare with when evaluating patients with SJS associated with acetaminophen intake. The median onset of SJS/TEN symptoms in the case studies reviewed was approximately 2-3 days after the ingestion of acetaminophen [8,10]. Other associated drugs such as anticonvulsants, allopurinol, and sulfonamide-based drugs were found to have a longer incubation period of up to four to 21 days before the onset of SJS/TEN when compared to antipyretic drugs [10]. In Nakamura et al. [8], a case report involving a six-year-old with the development of SJS with irreversible lung damage after initial suspicion of *Mycoplasma pneumoniae* was later discharged from the hospital with acetaminophen and clarithromycin. A drug lymphocyte stimulation test was ordered to rule out clarithromycin and confirm that acetaminophen was the causative agent after the onset of clinical symptoms of SJS. In two other case reports, a similar initial presentation of an upper respiratory infection was suspected then later returned with an adverse cutaneous and mucocutaneous reaction following the ingestion of acetaminophen [10].

Conversely, many studies attribute acetaminophen as the main culprit due to the shorter mean time of onset of the disease after ingestion of acetaminophen compared to other drug inducers that usually have a longer induced time. An analysis of the French database in Lebrun-Vignes et al. [11] using an algorithm of drug causality for epidermal necrolysis (ALDEN) concluded most cases found within the database have a confounding bias. The ALDEN scores were able to group each case into either acetaminophen being lower, equal to, or greater than the other susceptible drugs relating to induced SJS/TEN. Alternatively, this study raised suspicions about the inquiries provided in the database and only listed 12 out of the 112 case reports to have a “very probable” association that acetaminophen was the implication of drug causality for SJS/TEN [11].

One significant feature found within the last 15 years in multiple clinical studies involves the association between different HLA genotypes and SJS/TEN (Table 1). HLA-A 02:06 or HLA-B 44:03 are potential genetic risk factors for being more susceptible to implications of acetaminophen-related SJS/TEN (AR-SJS/TEN) with severe ocular complications (SOC). In one study, 57 of the 73 patients that reported taking acetaminophen before the onset of SJS or TEN with SOC were significantly associated with either HLA-A 02:06 or HLA-B 44:03 [12]. Other inducers of SJS/TEN such as allopurinol or carbamazepine were not strongly associated with SOC [13]. An international collaboration in genetically predisposed genotypes focused primarily on cases in Japan and included Thailand. These regions were selected for acetaminophen being the most frequently used OTC medication. Other international collaborators such as Brazil, India, China, and Korea were omitted due to listing their cold medicine-related SJS/TEN with SOC which often consisted of a mixture of multiple ingredients/medications therefore not pinpointing the main causative agent. In Japanese patients, the researchers analyzed the association with HLA-A 02:06 to be more strongly associated with AR-SJS/TEN with SOC than any other genotype noted. In Thai patients, the collaborators significantly associated the pathogenesis of AR-SJS/TEN with SOC when analyzing the HLA-A 33:03, HLA-B 44:03, or HLA-C 07:01. These high-risk populations suggested a synergistic effect with acetaminophen to down-regulate EP3 protein expression in the conjunctival epithelial cells leading to corneal defects [14]. EP3 is a receptor of PGE2 that negatively regulates mucosal inflammation and is found in lower expression levels in AR-SJS/TEN with SOC patients [13]. Therefore, it is vital to consider the implication of acetaminophen in certain countries due to the genetic factors that could lead to the possible risk of SJS/TEN.

**Table 1:** Several studies have associated common carrier frequencies of HLA genotypes seen in certain populations with acetaminophen-induced SJS/TEN. HLA: human leukocyte antigens

Table 1: HLA Genotypes Associated with Acetaminophen-Induced Stevens-Johnson Syndrome/Toxic Epidermal Necrolysis			
Author	Genotype carrier	Population Ethnicity	Results
Ueta et al. 2019 [15], Nakatani et al. 2019 [16]	HLA-A 02:06	Japanese	Strong association
Ueta et al. 2019 [15]	HLA-A 23:02	Japanese	Significant association
Ueta et al. 2019 [15], Nakatani et al. 2019 [16]	HLA-A 24:02	Japanese	Inverse association
Jongkhajornpong et al. 2022 [17]	HLA-A 33:03	Thai	Strong association
Ueta et al. 2019 [15]	HLA-B 13:01	Japanese	Significant association
Ueta et al. 2019 [15], Jongkhajornpong et al. 2022 [17]	HLA-B 44:03	Japanese Thai	Strong Association
Jongkhajornpong et al. 2022 [17]	HLA-C 07:01	Thai	Significant Association
Ueta et al. 2019 [15]	HLA-C 14:03	Japanese	Significant association
Ueta et al. 2019 [15]	HLA-DRB1	Japanese	No association
Ueta et al. 2019 [15]	HLA-DQB1	Japanese	No association

Therapeutic opinion

Acetaminophen: Indications for Use, Pharmacokinetics, Mechanism of Action, Adverse Effects

According to the FDA, the indicated uses of acetaminophen include use in pain and fever. It is also used in the manufacturing process of many other therapies related to allergy, cough, cold, flu, and insomnia [18]. Its use as a primary therapy is widespread and it also plays an important role as an adjuvant therapeutic. For patients that are at risk for GI ulcers or thrombopathies, acetaminophen is a useful medication as it is not contraindicated in these conditions. Effective dosing in acetaminophen usage, for pain and fever, is outlined by Mayo Clinic as 650-1000 mg every 4-6 hours for adults and teenagers. Children from age two to 12 have incrementally increasing doses that are indicated beginning with 160mg at age 2-4 and increasing by 80 mg every two years. Children under two years of age need to be evaluated by their physician before being administered acetaminophen [19]. The toxic dosing for acetaminophen is over 4,000 mg within 24 hours, as this can place one at significant risk for liver damage [19]. The hepatic damage that can occur from high doses of acetaminophen ultimately arises from pharmacokinetic interactions with P450 liver enzymes. After oral administration, peak plasma concentration occurs in approximately 90 minutes. Its plasma half-life ranges from 1.5 to 2.5 hours at the recommended dosages. In overdosed amounts, the plasma elimination rate is impaired and thus has been shown to extend plasma half-life to 4-8 hours [20]. Acetaminophen will be conjugated to glucuronic acid by UGT1A1, UGT1A6, UGT1A9, and UGT2B15. Acetaminophen will be sulfonated principally by SULT1A1 and SULT2A1. Small amounts of acetaminophen will be conjugated to form n-acetyl-p-benzoquinone-amine (NAPQI), which is the primary offender in hepatotoxicity when present in large amounts. The NAPQI will be reduced by glutathione (GSH), which will then be excreted in the urine as NAPQI-cysteine. As levels of GSH are depleted, the excess NAPQI will complex with mitochondrial proteins, leading to cell death [20]. The exact mechanism of action of acetaminophen has been infamously misunderstood for nearly a century. Given its widespread usage and high market valuation, more research in recent years has been performed to better understand this drug’s complex mechanism of action. Two landmark studies, performed by Zygmunt et al. [21] and Bertolini et al. [22], described the mechanism of acetaminophen, or paracetamol, to be more complex than previously appreciated. Paracetamol is converted to p-aminophenol and conjugated to form N-arachindonoylpheolamine, or AM404, a compound that acts as an endogenous cannabinoid. This compound acts as an agonist of TRPV1 and decreases cellular anandamide concentrations, leading to higher levels of endogenous cannabinoids which in turn act on cannabinoid receptors [22]. The role of agonists of cannabinoid receptors has been well associated with nociceptive dulling, anti-inflammatory, and synergistic effects with endogenous opioid receptors [23]. This is believed to be the primary mechanism by which acetaminophen induces its analgesic and antipyretic effects. The therapeutic benefits of acetaminophen usage are not without risk. Aside from the well-known liver damage that can occur at high doses, there are other complications of significant impact such as the association of acetaminophen with SJS and TEN.

Acetaminophen and Association with SJS and TEN

The occurrence of SJS and TEN are defined by the presence of widespread necrosis and sloughing of the skin [3,24]. Both are appropriately considered dermatologic emergencies and require immediate treatment [24]. More often women are affected by SJS/TEN and SJS occurs about three times more frequently than TEN [25]. Furthermore, the distinction between SJS/TEN is directly related to the total body surface area affected. Less than 10% of the total body surface area affected correlates to SJS, 10-30% denotes a malleable section shared by SJS/TEN, and any surface area >30% are diagnosed as TEN [26]. The initial reaction of these diseases is believed to be the result of a type IV hypersensitivity reaction due to potential drugs [27]. The initial symptoms will take about four to 21 days to occur, starting with a common prodrome of fever, influenza-like symptoms, lymphadenopathy, and discomfort swallowing in the first couple of days [28]. Coalescing, red macules with purpuric centers that develop into vesicles and bullae will begin to appear on the thorax and face, leading to skin sloughing in these areas before symmetrical movement to the rest of the body [28]. With lateral pressure applied by a clinician on the blisters, a positive Nikolsky sign, or separation of the intact superficial epidermis will help diagnose SJS/TEN [28,29]. Furthermore, almost all cases will be followed by a characteristic mucosal involvement, which usually is stomatitis and conjunctivitis [30]. Unfortunately, a diagnosis of SJS/TEN comes with significant consequences of deformity and death. One cohort study found the mortality rate to be 24% within six weeks, and that number rose to 34% within a year of the reaction [31]. More often than not, deaths within the first 90 days were commonly due to the severity of the reaction while deaths after 90 days started to relate to increased age and comorbidities [31].

The direct offending pathogenesis of SJS/TEN is still unclear; however, researchers have begun analyzing cytotoxic T lymphocytes and natural killer cells targeting either a drug that has not been modified or drugs presented through human leukocyte antigens on keratinocytes as a potential mechanism in SJS [32]. Multiple signals are released that create apoptosis of keratinocytes and subsequent damage of the skin, containing signs of increased CD8+ lymphocytes within the blisters that have drug-specific cytotoxicity in patients with TEN [33]. The peripheral tissues indicated higher signs of cytokine proliferation by IFN-γ, IL-2, IL-5, IL-6, IL-10, and IL-13, which are responsible for the proliferation and activation of T cells [34-37]. These specific findings present a more specific route for potential direct causes of cytotoxic T lymphocytes and natural killer cells, yet more recent research has begun to point to genetic predispositions of human leukocyte antigen. A study in a Chinese Han population correlated SJS to HLA-B15:02 as a genetic predisposition [38]. Furthermore, a study on a Japanese population discovered HLA-B58:01 as a genetic accomplice to SJS/TEN [39]. HLA-B15:02 was found to have a high likelihood of not being carbamazepine-related or aromatic anti-epileptic-related SJS/TEN while HLA-B58:01 had a high correlation with allopurinol-related SJS/TEN [39]. Similar genetic discoveries have not been found in European countries. These discoveries and current theories about SJS/TEN denote a strong component of both high-risk drugs and genetic dispositions.

While other drugs such as allopurinol and carbamazepine have been shown to be large contributors to SJS/TEN, physicians must be aware of the alarming percentage of cases caused by acetaminophen [40]. A recent nationwide survey of Korean patients with SJS/TEN showed that acetaminophen was the third largest direct contributor to SJS/TEN among patients, resulting in 5.2% of total SJS/TEN cases reported [40]. This correlation is not commonly mentioned in the literature and can lead physicians to not recognize the potential effects or causations of a patient with SJS/TEN. Furthermore, cases of SJS/TEN are usually rare, so more attention needs to be called to healthcare workers to recognize acetaminophen and its adverse effects [7]. Other associations have been made between specific genetic predispositions and acetaminophen-related SJS/TEN. One study examined 80 Japanese patients with acetaminophen-related SJS/TEN and ocular manifestations and found strong correlations to this hypersensitivity reaction with HLA-A02:06, HLA-B13:01, HLA-B44:03, and HLA-C14:03 while inverse correlations were noted with HLA-A24:02 [15]. Furthermore, another genotyping study on Thai patients with acetaminophen-related SJS/TEN found moderate to strong associations with HLA-A33:03 and HLA-C07:01 and interestingly found a strong association with HLA-B 44:03, which was a similar finding in the earlier study [15,17]. These findings, fortunately, present the foundations to explore genetic predispositions to acetaminophen-related SJS/TEN. All of the clinically associated HLA types will be further discussed in the future. While several associations have been found between different HLA types and acetaminophen-related SJS/TEN, more research is needed to analyze the potential implications of acetaminophen related to adverse effects. The therapy of the SJS/TEN has been recently reviewed, and cyclosporine seems the most efficacious therapy, especially if combined with intravenous immunoglobulin and corticosteroids [3,41]. Unfortunately, due to the rarity of the disease, there are no good prospective, randomized controlled trials. In any case, more studies are required analyzing genetic relationships and SJS/TEN caused by acetaminophen to properly understand and appreciate the full magnitude of risks associated with acetaminophen usage and to better define its potential therapy.

## Conclusions

Acetaminophen is an extremely effective OTC drug used for its antipyretic and analgesic effects. Its unique mechanism of action in comparison with other OTC drugs favors its use in pregnant patients. Collectively, these characteristics make acetaminophen incorporated into clinical practice globally. Except in the cases of an overdose, acetaminophen is relatively safe, allowing it to be used regularly. Moreover, 5.2% of SJS/TEN are attributed to acetaminophen-related reactions, making it the third largest contributor of SJS/TEN. While these cases do not commonly occur, physicians must be aware of the apparent risk in different individuals, especially those garnering genetic predispositions. Furthermore, some genetic variations in HLA types across different ethnicities have been shown to react with acetaminophen which can cause severe life-threatening adverse reactions. Although public opinion is extremely favorable for acetaminophen usage, it is imperative that patients and physicians alike recognize the risk associated with it. Awareness of these adverse afflictions can lead physicians to make more informed decisions to respond to SJS/TEN cases efficiently. Acetaminophen is a staple drug for antipyretic and analgesic effects, but physicians must consider the potential risks and outcomes to deliver thorough patient care and to quickly respond in the case of an emergency.
